# Dietary pectin enhances intestinal antimicrobial protein expression via a tuft cell–ILC2–STAT6 signaling axis

**DOI:** 10.1016/j.crfs.2026.101445

**Published:** 2026-05-20

**Authors:** Chisato Yanagi, Yoshiki Ishii, Shodai Ishikawa, Ryo Inoue, Dina Mustika Rini, Takuya Suzuki

**Affiliations:** a Graduate School of Integrated Sciences for Life, Hiroshima University, 1-4-4 Kagamiyama, Higashi-Hiroshima, 739-8528, Japan; b Faculty of Agriculture, Setsunan University, 45-1 Nagaotoge-cho, Hirakata, 573-0101, Japan

**Keywords:** Pectin, Tuft cell, ILC2, Antimicrobial protein, Intestinal immunity, Microbiota

## Abstract

Dietary fibers are known to modulate intestinal physiology; however, the cellular mechanisms linking dietary fiber intake to epithelial immune responses remain incompletely understood. Here, we investigated the effects of pectin, a citrus-derived dietary fiber, on antimicrobial protein expression in the mouse small intestine. Pectin supplementation markedly upregulated the expression of antimicrobial proteins, including RELMβ, ANG4, SPRR2A, and REG3 family members, accompanied by enrichment of gene pathways related to host defense. These effects were associated with activation of type 2 immune responses, as evidenced by increased expression of interleukin (IL)-25 and IL-13, expansion of tuft cells, and enhanced STAT6 phosphorylation. Pharmacological inhibition of group 2 innate lymphoid cells (ILC2) suppressed pectin-induced antimicrobial protein expression, indicating a critical role of ILC2-mediated signaling. Furthermore, these responses were completely abolished in tuft cell–deficient *Pou2f3*-knockout mice, demonstrating that tuft cells are indispensable for pectin-induced immune activation. In addition to host responses, pectin altered intestinal microbiota composition in both tuft cell–dependent and –independent manners, including a reduction in *Akkermansia* abundance under fiber-rich conditions. Collectively, these findings reveal that pectin activates a tuft cell–ILC2–STAT6 signaling axis to enhance antimicrobial protein production and modulate host–microbiota interactions. This study provides new insight into how dietary fibers directly regulate intestinal epithelial immunity and barrier function.

## Highlights

• Pectin enhances intestinal antimicrobial proteins via tuft–ILC2 signaling
• Tuft cell deficiency abolishes pectin-induced antimicrobial responses
• ILC2 inhibition suppresses pectin-induced STAT6 activation and AMPs
• Pectin alters microbiota in tuft-dependent and independent manners

## Graphical abstract

**Figure:**
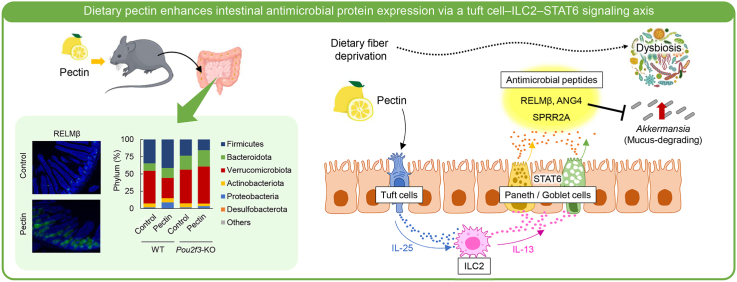

## 1 Introduction

Intestinal epithelium plays a central role in nutrient absorption while simultaneously acting as a physical and immunological barrier against luminal factors, including commensal and pathogenic microorganisms (Horowitz et al., 2023). This barrier function is maintained by multiple coordinated mechanisms, such as tight junctions, mucus layers, immunoglobulin A (IgA), and antimicrobial proteins (Neurath et al., 2025). Disruption of this barrier increases the translocation of bacterial components, such as lipopolysaccharides, into mucosal and systemic compartments, thereby promoting both local and systemic inflammation. Consequently, impaired intestinal barrier function is implicated in a wide range of diseases, including inflammatory bowel disease, metabolic disorders, liver diseases, chronic kidney disease, and pancreatitis (Neurath et al., 2025). Therefore, dietary strategies that reinforce intestinal barrier function have attracted increasing attention as potential approaches to support human health.

Intestinal antimicrobial proteins are key components of innate immunity that protect the host by limiting microbial invasion and shaping the composition of the gut microbiota (Chung et al., 2019). A diverse array of antimicrobial factors, including defensins, lysozymes, RegIII lectins (REG3), angiogenins, cathelicidins, resistin-like molecules (RELM), and small proline-rich proteins (SPRR2), contribute to mucosal defense (Propheter et al., 2017; Hu et al., 2021; Hooper et al., 2003; Vaishnava et al., 2011). In addition to their antimicrobial activity, these proteins play important roles in maintaining microbial homeostasis by modulating bacterial populations at the epithelial interface. Previous studies have demonstrated that various dietary components, such as dietary fibers, microbial metabolites (e.g., short-chain fatty acids), vitamins, and phytochemicals, can influence the expression of antimicrobial proteins (Wan et al., 2019). However, the cellular and molecular mechanisms underlying dietary regulation of antimicrobial protein production remain incompletely understood.

Dietary fibers exert diverse physiological effects in the gastrointestinal tract, traditionally attributed to microbial fermentation and the production of metabolites such as short-chain fatty acids (Koh et al., 2016). However, emerging evidence suggests that certain dietary fibers can also be directly sensed by intestinal epithelial cells, thereby regulating intestinal functions independently of the microbiota (Van Hung and Suzuki, 2017; Rini et al., 2023). Pectin, a major dietary fiber abundant in fruits, is a complex polysaccharide primarily composed of α-1,4-linked galacturonic acid residues, with structural variability depending on the degree of methyl esterification. Pectin has been reported to exhibit multiple beneficial effects, including anti-inflammatory activity and enhancement of intestinal barrier integrity (Jiang et al., 2025; Steigerwald et al., 2024). In addition to microbiota-dependent effects, pectin may directly interact with epithelial and immune cells, potentially through pattern-recognition receptors such as Toll-like receptors (Baggio et al., 2022; Jermendi et al., 2023). Nevertheless, how intestinal epithelial cells sense pectin and how this sensing is translated into functional host defense responses remain largely unknown.

The intestinal epithelium comprises multiple specialized cell types, including absorptive enterocytes, goblet cells, enteroendocrine cells, and tuft cells, which collectively maintain mucosal homeostasis. Tuft cells are a rare epithelial population (<1% of epithelial cells) that function as chemosensory sentinels of the intestinal lumen (Gerbe et al., 2016; Howitt et al., 2016). Upon sensing luminal stimuli such as helminths, tuft cells secrete mediators including interleukin-25 (IL-25) and acetylcholine, which activate group 2 innate lymphoid cells (ILC2s). Activated ILC2s produce interleukin-13 (IL-13), which acts on epithelial cells to induce epithelial remodeling and antimicrobial protein expression via STAT6 signaling (Gerbe et al., 2016; Howitt et al., 2016). Consistent with this epithelial–immune circuit, our previous study demonstrated that the dietary fiber psyllium enhances intestinal antimicrobial protein production through tuft cell–dependent mechanisms (Ishii et al., 2024). Despite these advances, whether and how dietary fibers such as pectin modulate tuft cell–mediated immune circuits remains unclear.

In the present study, we investigated the role of dietary pectin in the regulation of intestinal antimicrobial protein expression, focusing on its direct interaction with intestinal epithelial cells and the tuft cell–ILC2 signaling axis. Using wild-type and Pou2f3-deficient mice, we demonstrate that pectin enhances antimicrobial protein production through a tuft cell–dependent type 2 immune pathway, providing new insight into how dietary fibers modulate epithelial immunity and host–microbiota interactions.

## 2 Materials and methods

### 2.1 Pectin

Low-methoxyl pectin extracted from citrus peel was kindly provided by Tate & Lyle PLC (GENU® pectin type LM-102 AS-Z; London, UK). Based on GC–MS analyses (JMS-T2000GC AccuTOF GC-Alpha system; JEOL, Tokyo, Japan) described below, the monosaccharide composition of the pectin was rhamnose:galacturonic acid:galactose = 9.7:63.7:26.6 (Fig. S1), and the degree of esterification was 31.0% (Fig. S2).

### 2.2 Monosaccharide composition and degree of esterification of pectin

The monosaccharide composition and degree of esterification of pectin were determined by gas chromatography–mass spectrometry (GC–MS) as described previously (Akiyama et al., 2011). For monosaccharide analysis, 1% (w/v) pectin was hydrolyzed with 15% (v/v) trifluoroacetic acid. The released monosaccharides were converted to diethyldithioacetal derivatives using ethanethiol, followed by trimethylsilylation. Nine monosaccharides (xylose, arabinose, rhamnose, fucose, glucose, galactose, mannose, galacturonic acid, and glucuronic acid), which are commonly present in natural polysaccharides, were used as reference standards. Mannitol was used as an internal standard. Each derivatized monosaccharide was quantified using a JMS-T2000GC AccuTOF GC-Alpha system.

For determination of the degree of esterification, pectin was demethylated with 50 mmol/L NaOH, and the released methanol was quantified by GC–MS using the same system.

### 2.3 Animals

All animal experiments were approved by the Animal Care and Use Committee of Hiroshima University (approval no. C24-10). All procedures were conducted in accordance with the Guidelines for the Care and Use of Laboratory Animals at Hiroshima University, the National Research Council's Guide for the Care and Use of Laboratory Animals, and the ARRIVE guidelines. Seven-week-old female C57BL/6J mice were purchased from The Jackson Laboratory Japan (Yokohama, Japan). *Pou2f3*-knockout mice (*Pou2f3*-KO, RBRC05254) were obtained from RIKEN BRC through the National BioResource Project of MEXT/AMED, Japan (Matsumoto et al., 2011). *Pou2f3*-KO mice and wild-type (WT) littermates were generated by crossing heterozygous mice. Mice were housed under controlled conditions (22 ± 2 °C, 40–60% humidity, 12 h light/dark cycle with lights on from 08:00 to 20:00). Mice were acclimated for at least 1 week with free access to an AIN-93G-based fiber-free control diet (Table S1) and tap water. At the end of the experiments, mice were euthanized by exsanguination under isoflurane anesthesia. Only female mice were used to provide a consistent experimental background for evaluating the effect of pectin on small-intestinal immune responses. The study was designed as an initial mechanistic investigation under a single-sex condition.

### 2.4 Experimental design

Four feeding studies in mice (Experiments 1–4) were conducted to investigate the role of pectin in regulating intestinal antimicrobial protein expression. All animal experiments were performed in at least two independent experiments, and similar results were obtained. Unless otherwise indicated, the figures show data from one representative experiment.

In Experiment 1, the effect of pectin supplementation on gene expression profiles in the small intestine was examined. C57BL/6J mice (n = 14) were fed control or 10% (w/w) pectin diets for 5 days. Pectin was replaced with an equivalent amount of starch in the control diet. Jejunal tissue was subjected to whole transcriptome analysis by RNA sequencing, as described below. Jejunal tissues were selected for RNA-seq analysis because the jejunum has a relatively low bacterial load compared with distal intestinal regions, allowing us to evaluate host epithelial responses to dietary pectin with less influence from microbial fermentation. Jejunal, ileal, and colonic tissues were analyzed by qRT-PCR, immunoblotting, and immunofluorescence.

In Experiment 2, the effects of different doses of pectin on antimicrobial protein expression and type 2 immune responses were investigated. C57BL/6J mice (n = 28) were fed diets containing 2.5%, 5%, or 10% (w/w) pectin for 5 days. Pectin was replaced with an equivalent amount of starch in the control diet. Jejunal and ileal tissues were analyzed by qRT-PCR.

In Experiment 3, the role of ILC2 in pectin-induced antimicrobial protein expression was examined using disulfiram (DSF), a pharmacological inhibitor of ILC2. Mice (n = 28) were randomly assigned to four groups: control, pectin, control + DSF, and pectin + DSF. After acclimation, mice were fed control or 10% pectin diets for 5 days. Disulfiram (2.5 mg/kg body weight) was administered by oral gavage every 2 days prior to the start of the experimental diets. Control groups received ultrapure water. Small intestinal tissues (jejunum and ileum) were analyzed by qRT-PCR, immunoblotting, and immunofluorescence.

In Experiment 4, the role of tuft cells was examined using *Pou2f3*-deficient mice. Wild-type and *Pou2f3*-knockout mice (n = 14 per group) were fed control or 10% pectin diets for 5 days. Small intestinal tissues were analyzed by qRT-PCR, immunoblotting, and immunofluorescence. Small intestinal contents were collected for 16S rRNA gene-based microbiota analysis.

### 2.5 Whole transcriptome analysis of the mouse small intestine (experiment 1)

Total RNA was extracted from jejunal tissues using a NucleoSpin RNA kit (Macherey-Nagel, Düren, Germany) according to the manufacturer's instructions. RNA samples from each group (n = 7) were pooled and submitted to Genome-Lead for transcriptome sequencing using the DNBSEQ-G400S platform (PE150). Mapped read counts were normalized to transcripts per million (TPM). After adding 1, TPM values were log_2_-transformed and used to generate MA plots.

### 2.6 qRT-PCR analysis (experiments 1–4)

Total RNA was extracted using NucleoSpin RNA and Sepasol®-RNA I Super G (Nacalai Tesque, Kyoto, Japan) and reverse-transcribed into cDNA using a ReverTra Ace qPCR RT kit (TOYOBO, Osaka, Japan), according to the manufacturer's instructions. qRT-PCR was performed using SYBR Green Master Mix (Thermo Fisher Scientific, Waltham, MA, USA) on a StepOne Real-Time PCR System (Thermo Fisher Scientific). Primer sequences are listed in Table S2. Gene expression levels were calculated using the 2^−ΔΔCt^ method with ribosomal protein S28 (*Rps28*) as the reference gene.

### 2.7 Immunoblot analysis (experiments 1, 3, and 4)

Mouse small intestinal tissues (20 mg) were homogenized in 600 μL of lysis buffer [1% SDS, 1% Triton X-100, and 1% sodium deoxycholate in 30 mM Tris-HCl (pH 7.4) supplemented with protease and phosphatase inhibitors] using a Polytron homogenizer (KINEMATICA, Luzern, Switzerland). Protein concentrations were determined using a BCA Protein Assay kit (FUJIFILM Wako Chemicals, Osaka, Japan). Protein extracts were mixed with Laemmli sample buffer and heated at 95 °C for 10 min. Proteins (20 μg) were separated by SDS-PAGE and transferred onto PVDF membranes. After Ponceau S staining, membranes were blocked with skim milk and incubated with primary antibodies, followed by HRP-conjugated secondary antibodies. Detailed antibody information is provided in Table S3. Signals were detected using an enhanced chemiluminescence system (Western Lightning Plus-ECL, PerkinElmer) and visualized with an Amersham Imager 680 (Cytiva).

### 2.8 Immunofluorescence (experiments 1, 3, and 4)

Jejunal tissues were embedded in optimal cutting temperature compound (Sakura Finetek, Tokyo, Japan) and sectioned at 8 μm thickness. Sections were blocked with 5% normal goat and donkey serum and incubated with primary antibodies (anti-SPRR2A, ANG4, DCLK1, and RELMβ) at 4 °C overnight. Sections were then incubated with Alexa Fluor-conjugated secondary antibodies and DAPI for 1 h. Detailed antibody information is provided in Table S3. Fluorescence images were acquired using a Leica FW4000 fluorescence microscope (Leica Microsystems, Wetzlar, Germany).

### 2.9 16S rRNA gene sequencing and microbiota analysis (experiment 4)

Bacterial DNA was extracted from small intestinal contents using a NucleoSpin DNA Stool kit (Macherey-Nagel) according to the manufacturer's instructions. Library preparation and sequencing using the Illumina MiSeq platform were performed as described previously. The V3–V4 region of the 16S rRNA gene was amplified using primers 341F and 805R. Sequence data were processed using QIIME 2 (version 2024.5), with denoising performed using the DADA2 plugin. Amplicon sequence variants (ASVs) were assigned taxonomy using a sklearn classifier against the SILVA database (version 138). Singletons and sequences assigned to mitochondria or chloroplasts were removed. A phylogenetic tree was constructed using the SEPP algorithm.

### 2.10 Statistical analysis

Data are presented as mean ± s.e.m. Statistical analyses were performed using JMP Student Edition 19 (SAS Institute Inc., Cary, NC, USA). Data were first assessed for normality using the Shapiro–Wilk test and for homogeneity of variance using the Levene's test. For comparisons between two groups, Student's t-test was used when these assumptions were satisfied; otherwise, the Mann–Whitney *U* test was applied. For comparisons among three or more groups, one-way ANOVA followed by the Tukey–Kramer post hoc test was used for parametric data, whereas the Kruskal–Wallis test followed by the Steel–Dwass post hoc test was used for non-parametric data. Differences were considered statistically significant at *P* < 0.05.

## 3 Results

### 3.1 Pectin supplementation induces antimicrobial proteins and type 2 immune responses in the mouse small intestine (experiment 1)

**Fig. 1:**
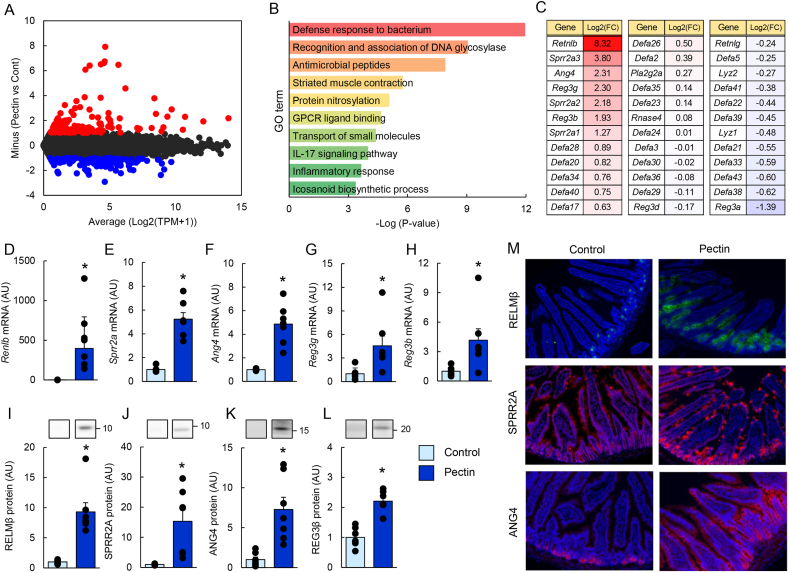
Pectin supplementation upregulates antimicrobial proteins in the mouse small intestine. Data are from Experiment 1. Mice were fed control or 10% pectin diets for 5 days. Jejunal tissues were subjected to RNA sequencing, and an MA plot based on log_2_-transformed transcripts per million (TPM) values is shown (A). “Average” indicates the mean expression level of the two groups, and “minus” represents the difference between groups. Each dot represents a gene; red and blue dots indicate genes upregulated (>2-fold) or downregulated (<0.5-fold), respectively, in the pectin group compared with the control group. Gene Ontology (GO) enrichment analysis of upregulated genes was performed using Metascape, and the top 10 GO terms ranked by *P* value are shown (B). Relative expression levels of antimicrobial protein genes are shown as fold changes compared with the control group (C). mRNA levels of *Retnlb*, *Sprr2a*, *Ang4*, *Reg3g*, and *Reg3b* were quantified by quantitative reverse transcription-PCR (qRT-PCR; D–H), and protein expression of RELMβ, SPRR2A, ANG4, and REG3β was analyzed by immunoblotting (E–M). Uncropped original immunoblot images are provided in Fig. S3. Representative immunofluorescence images of SPRR2A, RELMβ, and ANG4 in the jejunum are shown (F). Data are presented as mean ± s.e.m. Statistical significance was determined using Student's *t*-test or Mann–Whitney *U* test. *P* < 0.05 vs. control.

To examine the effect of pectin on intestinal gene expression, transcriptome analysis was performed in jejunal tissues of mice fed a pectin-containing diet. Jejunal tissues were selected for RNA-seq analysis because the jejunum contains a relatively low bacterial load compared with distal intestinal segments, allowing evaluation of host epithelial responses with less influence from microbial fermentation. Among 21,396 detected genes, 494 were upregulated and 591 were downregulated (>2-fold) in the 10% pectin group compared with controls (Fig. 1A). Gene ontology analysis revealed significant enrichment of pathways related to “defense response to bacterium,” “antimicrobial peptides,” and “inflammatory response” (Fig. 1B). Consistent with these results, the expression of antimicrobial protein genes, including *Retnlb*, *Sprr2a*, *Ang4*, *Reg3g*, and *Reg3b*, was significantly increased by pectin supplementation (Fig. 1C–H). Increased expression at the protein level was confirmed for RELMβ, SPRR2A, ANG4, and REG3β by immunoblotting (Fig. 1I–L, S3). Immunofluorescence analysis further demonstrated increased numbers of RELMβ-, SPRR2A-, and ANG4-positive cells in the jejunum (Fig. 1M), indicating an expansion of goblet and Paneth cell populations. REG3β was not detected by immunohistochemistry, likely due to antibody limitations.

**Fig. 2:**
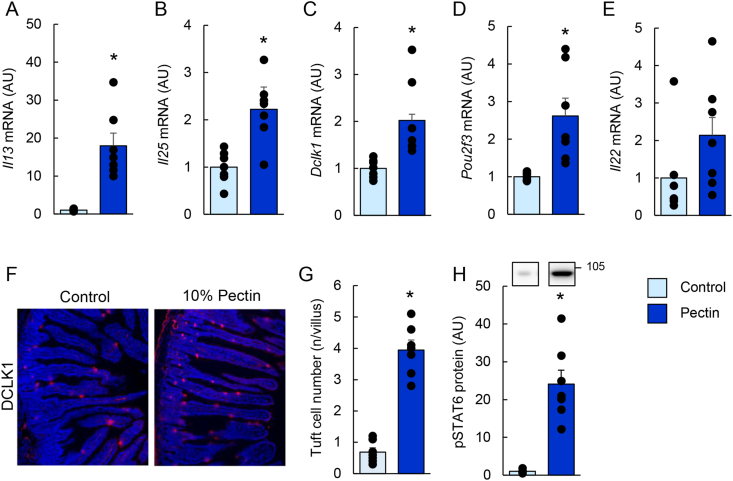
Pectin supplementation enhances type 2 immunity–related cytokines and expands tuft cells in the mouse small intestine. Data are from Experiment 1. Mice were fed control or 10% pectin diets for 5 days. mRNA expression levels of *Il13*, *Il25*, *Dclk1*, *Pou2f3*, and *Il22* were quantified by quantitative reverse transcription-PCR (qRT-PCR; A–E). Representative immunofluorescence images of DCLK1 staining in the jejunum are shown in (F), and the number of DCLK1-positive tuft cells is quantified in (G). Phosphorylation of STAT6 was analyzed by immunoblotting (H). Uncropped original immunoblot images are provided in Fig. S3. Data are presented as mean ± s.e.m. (n = 6). Statistical significance was determined using Mann–Whitney *U* test or Student's *t*-test. *P* < 0.05 vs. control.

Given that antimicrobial protein expression is regulated by type 2 immunity (Propheter et al., 2017; Hu et al., 2021; Hooper et al., 2003), we next examined related immune pathways. Pectin supplementation increased the mRNA expression of *Il13*, *Il25*, and tuft cell markers (*Dclk1* and *Pou2f3*), while *Il22* showed a non-significant trend toward increase (Fig. 2A–E). The number of DCLK1-positive tuft cells was also elevated (Fig. 2F and G), accompanied by increased STAT6 phosphorylation (Fig. 2H).

Similar effects were observed in the ileum, including increased expression of type 2 immunity-related antimicrobial proteins and associated cytokines, although REG3β protein expression was not significantly altered (Fig. S4A–C). In contrast, responses in the colon were less pronounced (Fig. S5).

These results indicate that dietary pectin increases the abundance of tuft cells, goblet cells, and Paneth cells in the small intestine, accompanied by enhanced expression of antimicrobial proteins and type 2 immunity-related markers. Because pectin-induced responses were evident in the jejunum and ileum but less pronounced in the colon in Experiment 1, subsequent analyses focused on the small intestine.

### 3.2 Pectin supplementation dose-dependently enhances type 2 immunity–related antimicrobial protein expression in the mouse small intestine (experiment 2)

**Fig. 3:**
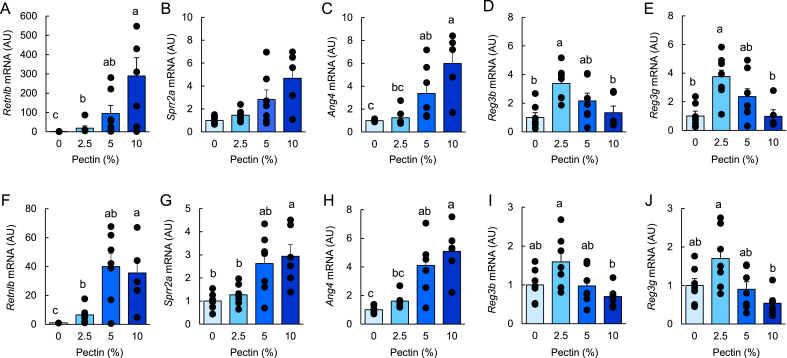
Pectin supplementation dose-dependently enhances antimicrobial protein expression in the mouse small intestine. Data are from Experiment 2. Mice were fed control or pectin-containing diets (2.5%, 5%, and 10%) for 5 days. mRNA expression levels of *Retnlb*, *Sprr2a*, *Ang4*, *Reg3g*, and *Reg3b* were quantified by quantitative reverse transcription-PCR (qRT-PCR) in the jejunum (A–E) and ileum (F–J). Data are presented as mean ± s.e.m. (n = 6). Statistical significance was determined using the Tukey–Kramer or Steel–Dwass test. Groups not sharing a common letter are significantly different (*P* < 0.05).

To determine the effective dose of pectin, mice were fed diets containing increasing concentrations of pectin. qRT-PCR analysis showed that the expression of type 2 immunity–associated antimicrobial proteins, including *Retnlb*, *Sprr2a*, and *Ang4*, was increased in a dose-dependent manner in both the jejunum and ileum (Fig. 3A–C, F–H), although the increase in *Sprr2a* in the jejunum did not reach statistical significance. Specifically, *Retnlb* expression was significantly elevated in the 2.5%, 5%, and 10% pectin groups, while *Ang4* expression was increased in the 5% and 10% groups. In contrast, the expression of *Reg3b* and *Reg3g* showed a distinct pattern, with the highest levels observed in the 2.5% pectin group in the jejunum (Fig. 3D, E, I, J).

These results indicate that pectin induces antimicrobial protein expression in a dose-dependent manner, while REG3 family genes exhibit a distinct, non-linear response to pectin supplementation.

### 3.3 ILC2 mediates pectin-induced antimicrobial protein expression in the mouse small intestine (experiment 3)

**Fig. 4:**
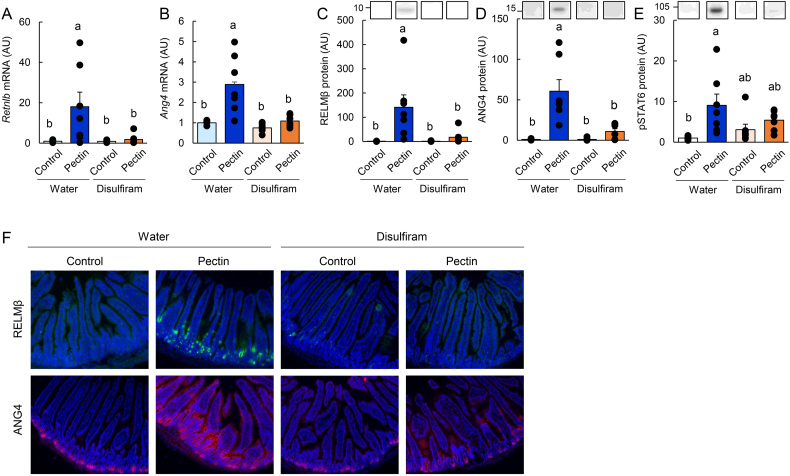
ILC2 mediates pectin-induced antimicrobial protein expression in the mouse small intestine. Data are from Experiment 3. Mice were fed control or pectin-containing diets with or without disulfiram for 5 days. Jejunal mRNA expression levels of *Retnlb* and *Ang4* were quantified by qRT-PCR (A, B). Protein expression of RELMβ, ANG4, and phosphorylated STAT6 was analyzed by immunoblotting (C–E). Uncropped original immunoblot images are provided in Fig. S6. Representative immunofluorescence images of RELMβ and ANG4 in the jejunum are shown (F). Statistical significance was determined using the Tukey–Kramer or Steel–Dwass test. Groups not sharing a common letter are significantly different (*P* < 0.05).

**Fig. 5:**
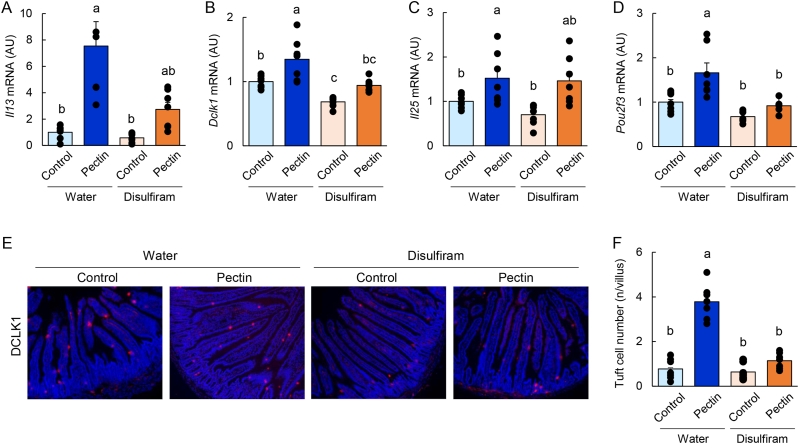
ILC2 mediates pectin-induced type 2 immune responses and tuft cell expansion in the mouse small intestine. Data are from Experiment 3. Mice were fed control or pectin-containing diets with or without disulfiram for 5 days. Jejunal mRNA expression levels of *Il13*, *Il25*, *Dclk1*, and *Pou2f3* were quantified by qRT-PCR (A–D). Representative immunofluorescence images of DCLK1 staining in the jejunum are shown in (E), and the number of DCLK1-positive tuft cells is quantified in (F). Statistical significance was determined using the Tukey–Kramer or Steel–Dwass test. Groups not sharing a common letter are significantly different (*P* < 0.05).

To determine the involvement of ILC2s in pectin-mediated antimicrobial protein expression, mice were treated with disulfiram, a pharmacological inhibitor of ILC2 activity. As expected, pectin supplementation increased the mRNA and protein expression of *Retnlb* and *Ang4* in the jejunum (Fig. 4A–D, S6). This effect was largely abolished by disulfiram treatment, whereas disulfiram alone had minimal effects under control diet conditions. Consistently, disulfiram suppressed pectin-induced STAT6 phosphorylation in the jejunum (Fig. 4E–S6) and reduced the number of RELMβ- and ANG4-positive epithelial cells (Fig. 4F). Furthermore, disulfiram treatment attenuated pectin-induced upregulation of *Il13*, *Dclk1*, and *Pou2f3* expression, as well as the expansion of tuft cells (Fig. 5A–F).

Similar inhibitory effects were observed in the ileum, where disulfiram prevented pectin-induced increases in *Retnlb*, *Ang4*, *Dclk1*, *Il13*, *Il25*, and *Pou2f3* expression (Fig. S7).

These results demonstrate that pectin-induced antimicrobial protein expression is mediated by ILC2-dependent type 2 immune signaling.

### 3.4 Tuft cells are essential for pectin-induced antimicrobial protein expression in the mouse small intestine (experiment 4)

**Fig. 6:**
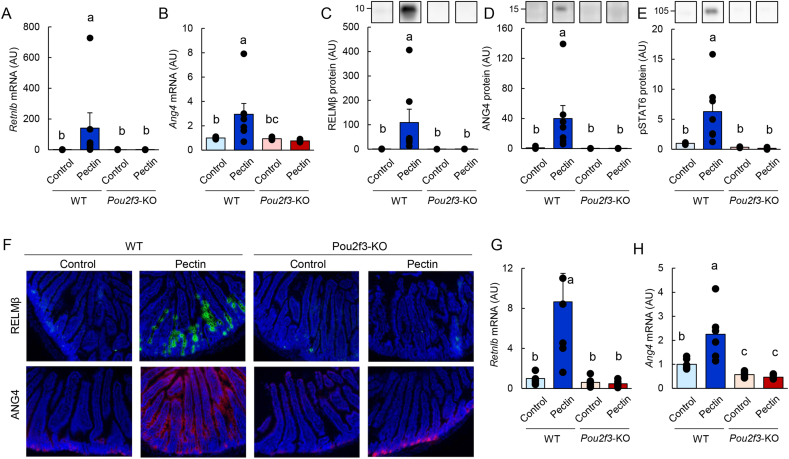
Tuft cells are essential for pectin-induced antimicrobial protein expression in the mouse small intestine. Data are from Experiment 4. Wild-type and *Pou2f3*-knockout mice were fed control or pectin-containing diets for 5 days. Jejunal mRNA expression levels of *Retnlb* and *Ang4* were quantified by qRT-PCR (A, B). Protein expression of RELMβ, ANG4, and phosphorylated STAT6 was analyzed by immunoblotting (C–E). Uncropped original immunoblot images are provided in Fig. S8. Representative immunofluorescence images of RELMβ and ANG4 in the jejunum are shown (F). Ileal mRNA expression levels of *Retnlb* and *Ang4* were quantified by qRT-PCR (G, H). Statistical significance was determined using the Tukey–Kramer or Steel–Dwass test. Groups not sharing a common letter are significantly different (*P* < 0.05).

**Fig. 7:**
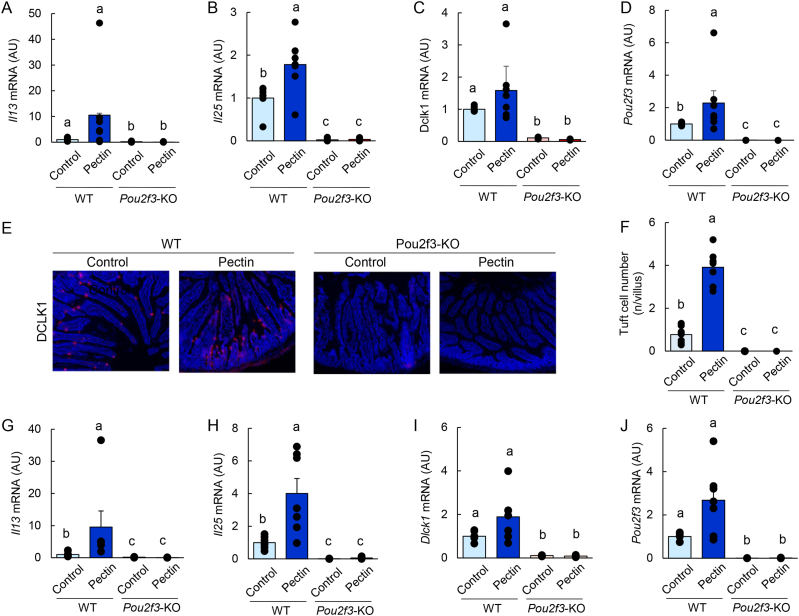
Tuft cells are essential for pectin-induced type 2 immune responses and tuft cell expansion in the mouse small intestine. Data are from Experiment 4. Wild-type and *Pou2f3*-knockout mice were fed control or pectin-containing diets for 5 days. Jejunal mRNA expression levels of *Il13*, *Il25*, *Dclk1*, and *Pou2f3* were quantified by qRT-PCR (A–D). Representative immunofluorescence images of DCLK1 staining in the jejunum are shown in (E), and the number of DCLK1-positive tuft cells is quantified in (F). Ileal mRNA expression levels of *Il13*, *Il25*, *Dclk1*, and *Pou2f3* were quantified by qRT-PCR (G–J). Statistical significance was determined using the Tukey–Kramer or Steel–Dwass test. Groups not sharing a common letter are significantly different (*P* < 0.05).

To determine the role of tuft cells in pectin-mediated responses, tuft cell–deficient *Pou2f3*-knockout mice and wild-type controls were fed a pectin-containing diet. In wild-type mice, pectin supplementation increased the mRNA and protein expression of RELMβ and ANG4, along with enhanced STAT6 phosphorylation in the jejunum (Fig. 6A–E, S8). In contrast, these effects were completely abolished in *Pou2f3*-knockout mice. Immunofluorescence analysis further confirmed that pectin-induced expansion of RELMβ- and ANG4-positive epithelial cells was absent in the jejunum of *Pou2f3*-knockout mice (Fig. 6F). Similarly, pectin failed to increase RELMβ and ANG4 expression in the ileum of knockout mice (Fig. 6G and H). Consistent with these findings, pectin did not induce the expression of *Il13*, *Il25*, *Dclk1*, or *Pou2f3*, nor did it increase the number of DCLK1-positive tuft cells in either the jejunum or ileum of *Pou2f3*-knockout mice (Fig. 7A–J).

These results demonstrate that tuft cells are indispensable for pectin-induced activation of type 2 immune signaling and subsequent antimicrobial protein expression in the small intestine.

### 3.5 Pectin modulates intestinal microbiota composition in both tuft cell–dependent and –independent manners (experiment 4)

**Fig. 8:**
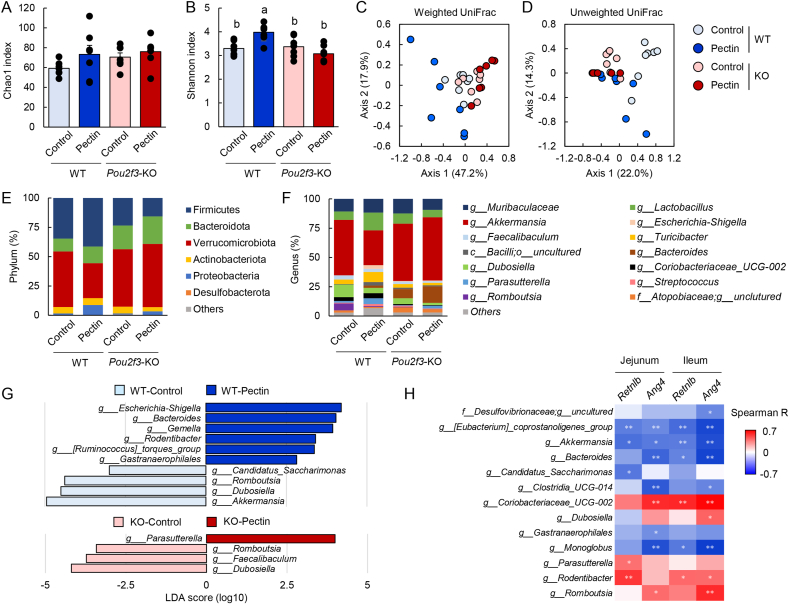
Pectin supplementation alters microbiota composition in the mouse small intestine. Data are from Experiment 4. Wild-type and *Pou2f3*-knockout mice were fed control or pectin-containing diets for 5 days. Small intestinal microbiota were analyzed by 16S rRNA gene sequencing. Alpha diversity was assessed using the Chao1 index (A) and Shannon index (B). Beta diversity was evaluated by principal coordinate analysis based on weighted and unweighted UniFrac distances (C, D). Relative abundances of microbiota at the phylum (E) and genus (F) levels are shown. Differentially abundant taxa were identified by LEfSe analysis (G). Correlations between bacterial genera and antimicrobial protein expression in the jejunum and ileum were analyzed using Spearman's correlation (H). Data are presented as mean ± s.e.m. (n = 8). Statistical significance was determined using the Tukey–Kramer or Steel–Dwass test. Groups not sharing a common letter are significantly different (*P* < 0.05).

Given the role of antimicrobial proteins in shaping the gut microbiota (Chung et al., 2019), we next examined the impact of pectin on microbial composition. Alpha diversity analysis revealed that pectin supplementation increased the Shannon index in wild-type mice but not in *Pou2f3*-knockout mice, whereas the Chao1 index was unaffected by either genotype or diet (Fig. 8A and B). These results suggest that pectin enhances microbial evenness in a tuft cell–dependent manner. Beta diversity analysis using UniFrac distances demonstrated significant differences in microbial community structure among groups (Fig. 8C and D). Notably, differences between control and pectin-fed groups were less pronounced in *Pou2f3*-knockout mice, indicating a partial dependence on tuft cells (Table S4 and S5). At the phylum level, pectin altered the relative abundance of major bacterial taxa, including a reduction in Verrucomicrobiota specifically in wild-type mice (Fig. 8E–S9). Consistently, the abundance of *Akkermansia*, a dominant genus within this phylum, was decreased by pectin in wild-type mice but not in *Pou2f3*-knockout mice (Fig. 8F–S10, S11), suggesting tuft cell–dependent regulation. LEfSe analysis further identified multiple taxa such as *Romboutsia* and *Dubosiella* altered by pectin supplementation in both genotypes (Fig. 8G), indicating that pectin also exerts tuft cell–independent effects on microbiota composition. Correlation analysis revealed that several bacterial genera were associated with the expression levels of antimicrobial proteins, including negative correlations for *Akkermansia* and positive correlations for *Rodentibacter* (Fig. 8H).

These results suggest that pectin modulates gut microbiota composition through both tuft cell–dependent mechanisms, likely mediated by antimicrobial proteins, and tuft cell–independent pathways.

## 4 Discussion

Intestinal barrier integrity is essential for preventing the translocation of luminal inflammatory molecules into host tissues, and its disruption contributes to both intestinal and systemic disorders (Horowitz et al., 2023; Neurath et al., 2025). In the present study, we demonstrate that dietary pectin enhances the expression of antimicrobial proteins in the mouse small intestine, thereby reinforcing epithelial barrier function. Notably, our findings suggest that pectin can directly interact with intestinal epithelial cells, rather than acting solely through microbiota-derived metabolites. Mechanistically, pectin activates tuft cells, leading to IL-25 production and subsequent activation of ILC2. IL-13 produced by ILC2 promotes antimicrobial protein expression, including RELMβ and ANG4, via STAT6 signaling. In addition, these antimicrobial proteins contribute to shaping intestinal microbiota composition. Together, our results reveal a novel epithelial–immune–microbiota axis through which dietary fiber regulates intestinal homeostasis.

A diverse repertoire of epithelial-derived antimicrobial proteins is critical for maintaining host–microbial segregation and preventing pathogenic invasion. For instance, RELMβ deficiency increases susceptibility to bacterial invasion and colitis (Hogan et al., 2006), while ANG4 exhibits antibacterial activity against both commensal and pathogenic bacteria (Hooper et al., 2003; Sultana et al., 2022). Moreover, reduced REG3 expression is associated with dysbiosis and increased intestinal permeability (Loonen et al., 2014; Frazier et al., 2022). These findings highlight the importance of antimicrobial protein regulation as a potential strategy to improve intestinal and systemic health.

Our data provide strong evidence that tuft cells and ILC2 are essential mediators of pectin-induced antimicrobial responses in the small intestine. Tuft cells act as luminal chemosensory sentinels that initiate type 2 immune responses through the secretion of IL-25, which subsequently activates ILC2 (Gerbe et al., 2016; Howitt et al., 2016). In the present study, pectin-induced upregulation of antimicrobial proteins was completely abolished in *Pou2f3*-deficient mice and markedly attenuated by pharmacological inhibition of ILC2, demonstrating a causal role of the tuft cell–ILC2 axis in this process. Furthermore, pectin supplementation enhanced the expression of IL-13 and promoted STAT6 phosphorylation in intestinal epithelial cells, consistent with activation of downstream type 2 immune signaling (von Moltke et al., 2016). This signaling cascade was accompanied by expansion of tuft and goblet cell populations, suggesting that pectin not only regulates antimicrobial protein expression at the transcriptional level but also induces epithelial remodeling. Taken together, these findings indicate that dietary pectin activates a coordinated epithelial–immune circuit, in which tuft cells initiate ILC2-mediated responses that drive both antimicrobial defense and epithelial adaptation, thereby contributing to the maintenance of intestinal barrier function. However, because pharmacological inhibition may have off-target effects, the results obtained with disulfiram should be interpreted with caution. Future studies using more specific genetic approaches, such as ILC2-deficient mice, will be needed to confirm the precise contribution of ILC2s to pectin-induced intestinal responses.

Pectin also modulated intestinal microbiota composition through both tuft cell–dependent and –independent mechanisms. Notably, pectin supplementation reduced the abundance of *Akkermansia*, a mucin-degrading bacterium (Derrien et al., 2004), in a tuft cell–dependent manner. This finding should be interpreted in the context of the control diet used in this study. Fiber-free diets are known to promote dysbiosis by reducing fiber-degrading bacteria and increasing mucin-degrading bacteria such as *Akkermansia*, as these microbes shift toward host-derived glycans as their primary energy source (Desai et al., 2016). In contrast, pectin supplementation likely restored the availability of dietary substrates for fiber-degrading bacteria, thereby reducing the reliance on mucin degradation and decreasing *Akkermansia* abundance. Interestingly, this pectin-induced reduction of *Akkermansia* was abolished in *Pou2f3*-deficient mice, indicating that tuft cells are required for this microbial shift. These findings suggest that, in addition to substrate-driven ecological changes, tuft cell–dependent antimicrobial responses may contribute to the regulation of mucin-degrading bacteria under fiber-rich conditions. In addition to *Akkermansia*, pectin altered the abundance of several other bacterial genera, including increases in *Bacteroides* and *Parasutterella*, and reductions in *Dubosiella* and *Romboutsia*, indicating both tuft cell–dependent and –independent modulation of the microbiota.

Interestingly, REG3 expression exhibited a distinct dose-response pattern compared with type 2 immunity–related antimicrobial proteins. While RELMβ and ANG4 increased in a dose-dependent manner, REG3 expression peaked at lower pectin concentrations. Because REG3 is primarily regulated by IL-22 derived from ILC3 (Zheng et al., 2008), whereas RELMβ and ANG4 are induced by IL-13 from ILC2 (Artis et al., 2004), this divergence may reflect differential activation of immune pathways depending on pectin dosage. It is possible that higher doses of pectin preferentially activate ILC2-mediated type 2 immunity, which in turn may suppress or counterbalance ILC3 activity, as reported in certain inflammatory and helminth-associated contexts (Hu et al., 2021). Consistent with this notion, the robust induction of IL-13 and STAT6 signaling observed in this study may contribute to the attenuation of REG3 expression at higher pectin concentrations. These findings suggest that dietary fiber can differentially regulate antimicrobial protein families through distinct immune pathways, potentially balancing ILC2 and ILC3 responses in the intestinal environment.

Several limitations of this study should be acknowledged. First, although our findings demonstrate that pectin-induced antimicrobial responses require tuft cells and ILC2s, the mechanism linking pectin to tuft cell activation remains unresolved. In particular, the present data do not provide direct evidence that pectin itself is sensed by intestinal epithelial cells or directly activates tuft cells. An alternative possibility is that pectin indirectly promotes tuft cell–associated immune responses by altering the gut microbiota and/or microbial metabolites in the intestinal lumen. For example, changes in luminal factors such as succinate, short-chain fatty acids, or other microbiota-derived signals may contribute to the observed responses. Tuft cells are known to detect luminal stimuli through G protein–coupled receptors (GPCRs), such as Sucnr1 and taste receptors (Nadjsombati et al., 2018; Xiong et al., 2022); however, whether similar receptors or signaling pathways are involved in pectin-induced responses remains to be determined. Further studies using germ-free mice, antibiotic-treated mice, or reductionist epithelial systems will be needed to distinguish direct from microbiota-mediated mechanisms and to clarify how dietary pectin engages tuft cell–associated signaling. Second, this study utilized a single type of citrus-derived pectin, and the physicochemical properties of pectin, including the degree of methyl esterification and monosaccharide composition, can vary depending on the source and processing methods. Such structural variations may influence its biological activity and interaction with epithelial and immune cells. Therefore, it remains to be determined whether the observed effects are specific to this particular pectin or represent a more general property of pectins. Third, this study did not directly assess whether pectin itself exerts antimicrobial activity. Although our data support the idea that pectin enhances host antimicrobial responses through the tuft cell–ILC2 axis, pectin also altered microbial community structure, including changes that were partly independent of tuft cells. Therefore, we cannot exclude the possibility that some of the observed microbiota changes were influenced not only by host-mediated antimicrobial responses but also by direct effects of pectin on intestinal bacteria. Further studies, including direct in vitro evaluation of the antimicrobial activity of pectin, will be needed to distinguish host-mediated effects from potential direct actions of pectin on the microbiota. Fourth, only female mice were used; therefore, potential sex-dependent differences in the response to dietary pectin were not evaluated. Future studies including male mice will be needed to determine the generalizability of the present findings. Finally, while our data suggest that antimicrobial protein–mediated mechanisms contribute to microbiota modulation, the causal relationships between specific microbial changes and host physiological outcomes were not directly assessed. Future studies integrating functional microbiome analyses and host response evaluations will be required to further clarify these interactions.

In conclusion, this study demonstrates that dietary pectin enhances intestinal antimicrobial protein expression through a tuft cell–ILC2–STAT6 signaling axis. This epithelial–immune interaction contributes to the regulation of intestinal microbiota composition and barrier function. Our findings provide new insight into how dietary fibers directly modulate intestinal immunity and highlight tuft cells as a key interface linking diet to host defense mechanisms.

## Author contributions

Chisato Yanagi: Investigation, Data curation, Formal analysis, Visualization.

Yoshiki Ishii: Investigation, Methodology, Formal analysis.

Shodai Ishikawa: Investigation, Data curation.

Ryo Inoue: Methodology, Supervision, Formal analysis, Writing – review & editing.

Dina Mustika Rini: Investigation, Methodology, Formal analysis, Writing – review & editing.

Takuya Suzuki: Conceptualization, Supervision, Funding acquisition, Project administration, Writing – original draft, Writing – review & editing.

## Declaration of competing interest

The authors declare that they have no known competing financial interests or personal relationships that could have appeared to influence the work reported in this paper.

## Data Availability

Data will be made available on request.
