# Supplementary material for: Dietary pectin enhances intestinal antimicrobial protein expression via a tuft cell–ILC2–STAT6 signaling axis
Source: Curr Res Food Sci. 2026 May 20;12:101445. doi: 10.1016/j.crfs.2026.101445 (PMC13234251; doi:10.1016/j.crfs.2026.101445)
Supplement: Multimedia component 1 [file mmc1.pdf]

**Table S1. Diet compositions in Experiments 1–4**

| Ingredients | Control | 2.5% Pectin | 5% Pectin | 10% Pectin |
|---|---|---|---|---|
| | g/kg diet | | | |
| Starch[1] | 579.5 | 554.5 | 529.5 | 479.5 |
| Sucrose[2] | 100.0 | 100.0 | 100.0 | 100.0 |
| Casein[3] | 200.0 | 200.0 | 200.0 | 200.0 |
| L-cysteine | 3.0 | 3.0 | 3.0 | 3.0 |
| Soybean oil | 70.0 | 70.0 | 70.0 | 70.0 |
| Mineral mix[4] | 35.0 | 35.0 | 35.0 | 35.0 |
| Vitamin mix[4] | 10.0 | 10.0 | 10.0 | 10.0 |
| Choline bitrate | 2.5 | 2.5 | 2.5 | 2.5 |
| Pectin[5] | 0.0 | 25.0 | 50.0 | 100.0 |
| Total | 1000.0 | 1000.0 | 1000.0 | 1000.0 |

[1] Starch (Chuo-syokuryou Industry, Inazawa, Japan).

[2] Sucrose (Nissin Sugar, Tokyo, Japan).

[3] Casein (ALACID; New Zealand Daily Board, Wellington, New Zealand).

[4] Mineral and vitamin mixtures were prepared according to the AIN-93G formulation.

[5] GENU® pectin type LM-102 AS-Z (Tate & Lyle PLC)

**Table S2. Primers for qRT-PCR analysis used in this study**

| Target genes | Forward (5′ to 3′) | Reverse (5′ to 3′) |
|---|---|---|
| Mouse *Sprr2a* | TCTTCCTTCAGTGTGGCCTG | CACAGGAGGGCATGTTGACT |
| Mouse *Retnlb* | TCTCAGTCGTCAAGAGCCTAA | GCCACAAGCACATCCAGTGA |
| Mouse *Ang4* | ACCACTTGTACGCACTCAGG | TTAAAGGCTCGGTACCCGCA |
| Mouse *Reg3b* | ATATACCCTCCGCACGCATTAG | AGGCCAGTTCTGCATCAAAC |
| Mouse *Reg3g* | TGCTTCCCCGTATAACCATCAC | TTGGCAACTTCACCTTGCAC |
| Mouse *Il13* | CACACAAGACCAGACTCCCC | GTTGGTCAGGGAATCCAGGG |
| Mouse *Il25* | CCTTGGAGCTATGAGTTGGACA | TGTGGGAGCCTGTCTGTAGG |
| Mouse *Pou2f3* | TGTGCAAACTCAAGCCACTG | TCTTCCTGCCAAACACTTCG |
| Mouse *Rps28* | ATCAAGCTGGCTAGGGTAACC | GGCCTTTGACATTTCGGATGA |

**Table S3. Details of antibodies used in this study**

| Antibodies | Source | Catalog Number | Immunoblot | Immunofluorescence |
|---|---|---|---|---|
| Rabbit anti-SPRR2A | Sigma-Aldrich | Custom-made | 1:4000 | 1:200 |
| Goat anti-RELMB | R & D System | AF2730 | 1:2000 | 1:200 |
| Rabbit anti-ANG4 | CUSABIO Tech. | PA661010ZA01MO | 1:10000 | 1:600 |
| Rabbit anti pSTAT6 | Cell Signaling Tech. | 56554 | 1:6000 | 1:200 |
| Rabbit anti- DCLK1 | Abcam | ab31704 | --- | 1:1000 |
| Goat anti-rabbit IgG-HRP | SeraCare | 074-1506 | 1:20000 | --- |
| Goat anti-mouse IgG-HRP | SeraCare | 074-1806 | 1:20000 | --- |
| Goat anti-rabbit IgG-AlexaFluor488 | Abcam | ab150077 | --- | 1:200 |
| Donkey anti-goat IgG-AlexaFluor488 | Abcam | ab150133 | --- | 1:200 |

**Table S4. Results of pairwise PERMANOVA based on weighted UniFrac distances**

| | | | pseudo-F | q-value |
|---|---|---|---|---|
| WT-Cont | vs | WT-Pectin | 4.01 | 0.002 |
| WT-Cont | vs | KO-Cont | 5.00 | 0.002 |
| WT-Cont | vs | KO-Pectin | 10.27 | 0.002 |
| WT-Pectin | vs | KO-Cont | 4.93 | 0.007 |
| WT-Pectin | vs | KO-Pectin | 8.56 | 0.005 |
| KO-Cont | vs | KO-Pectin | 2.77 | 0.030 |

**Table S5. Results of pairwise PERMANOVA based on unweighted UniFrac distances**

| | | | pseudo-F | q-value |
|---|---|---|---|---|
| WT-Cont | vs | WT-Pectin | 3.09 | 0.005 |
| WT-Cont | vs | KO-Cont | 3.82 | 0.005 |
| WT-Cont | vs | KO-Pectin | 5.86 | 0.005 |
| WT-Pectin | vs | KO-Cont | 2.52 | 0.005 |
| WT-Pectin | vs | KO-Pectin | 2.26 | 0.012 |
| KO-Cont | vs | KO-Pectin | 1.46 | 0.076 |
