# Supplementary material for: Dietary pectin enhances intestinal antimicrobial protein expression via a tuft cell–ILC2–STAT6 signaling axis
Source: Curr Res Food Sci. 2026 May 20;12:101445. doi: 10.1016/j.crfs.2026.101445 (PMC13234251; doi:10.1016/j.crfs.2026.101445)
Supplement: Multimedia component 2 [file mmc2.pdf]

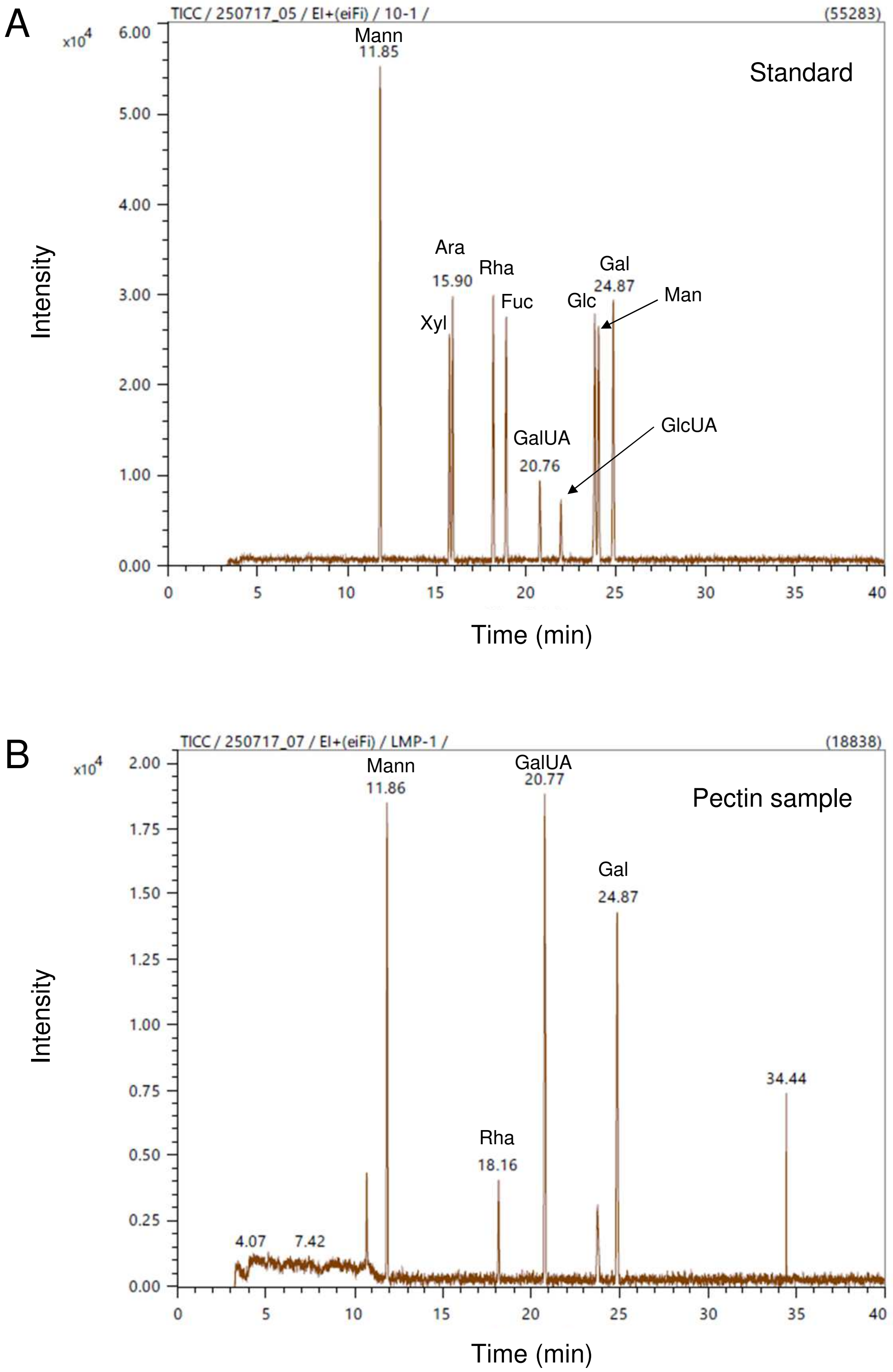

Fig. S1. Monosaccharide composition of pectin analyzed by gas chromatography–mass spectrometry.

The monosaccharide composition of pectin was analyzed by gas chromatography–mass spectrometry (GC–MS) following acid hydrolysis and derivatization. Representative chromatograms of standard monosaccharides (A) and pectin (B) are shown. Nine monosaccharides (xylose, arabinose, rhamnose, fucose, glucose, galactose, mannose, galacturonic acid, and glucuronic acid) were used as references.

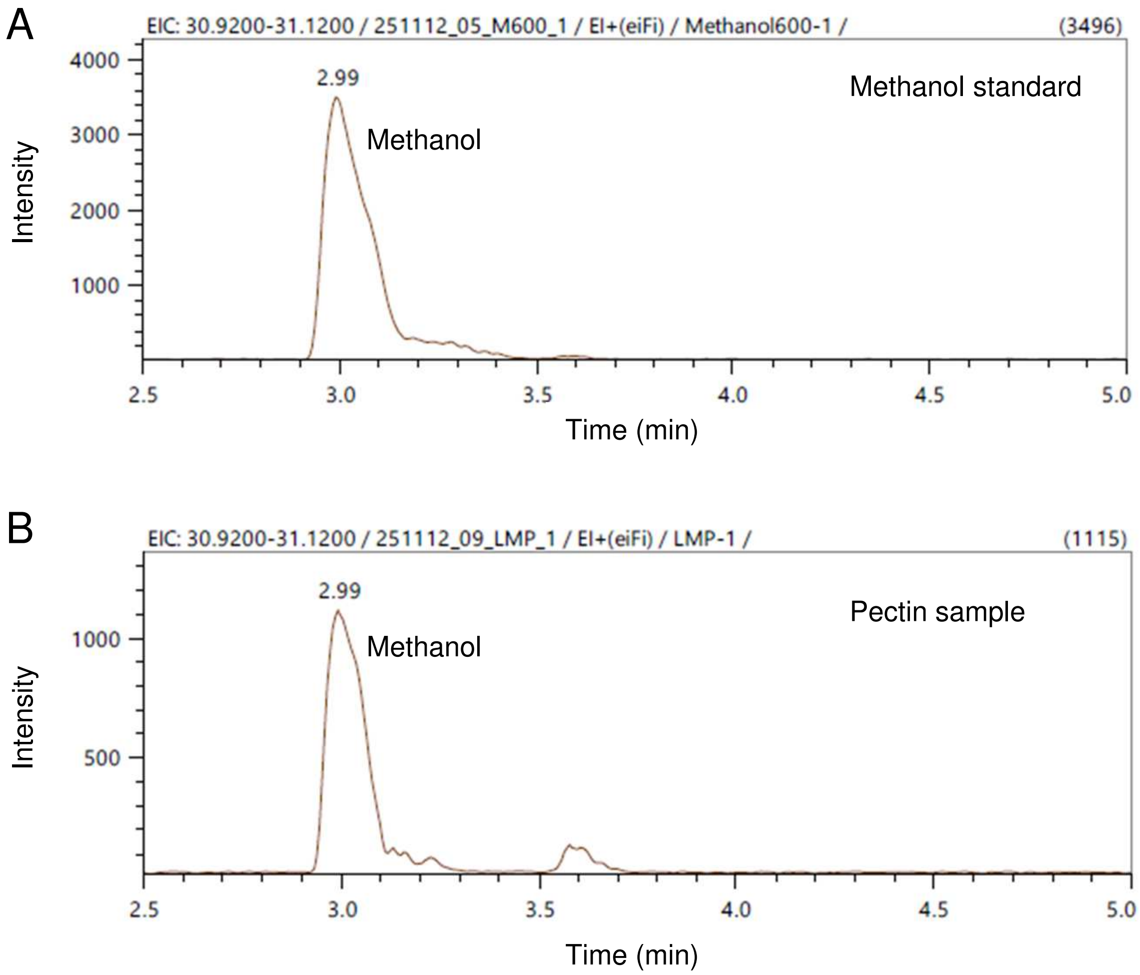

Fig. S2. Determination of the degree of esterification of pectin by gas chromatography–mass spectrometry.

The degree of esterification of pectin was determined by gas chromatography–mass spectrometry (GC–MS) based on methanol released after alkaline demethylation. Representative chromatograms of methanol standard (A) and methanol derived from pectin (B) are shown.

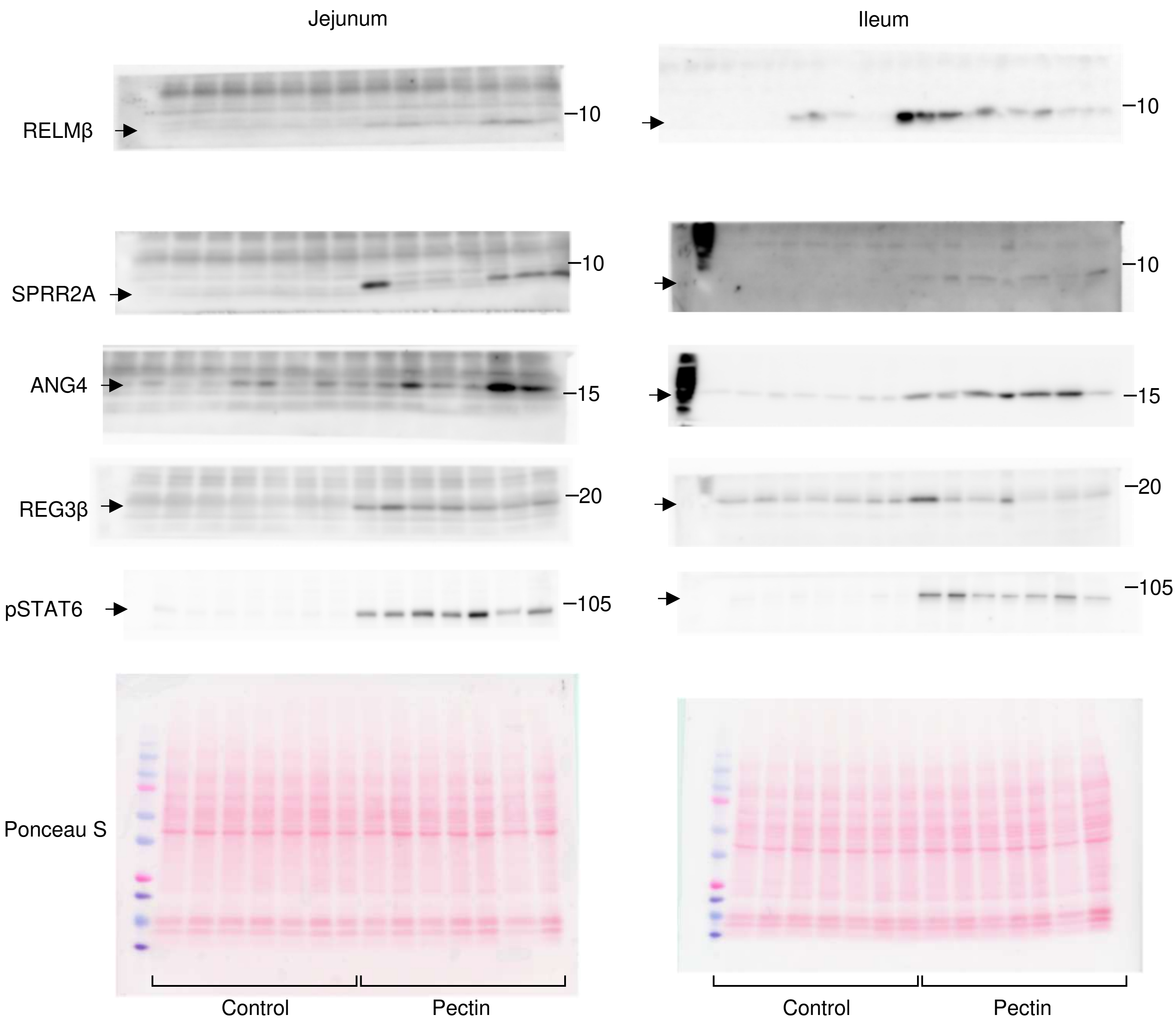

Fig. S3. Uncropped original immunoblot images corresponding to the blots shown in Fig. 1, 2, and S4.
Data are from Experiment 1. Mice were fed control or 10% pectin diets for 5 days. Jejunal and ileal expression of RELMβ, SPRR2A, ANG4, REG3β, and pSTAT6 was analyzed by immunoblotting. After transfer to PVDF membranes, total protein loading was visualized by Ponceau S staining and used for normalization. Membranes were cut horizontally according to molecular weight markers and probed separately with primary antibodies. The sample marked with an asterisk is unrelated to the present experiment and was not included in the analysis.

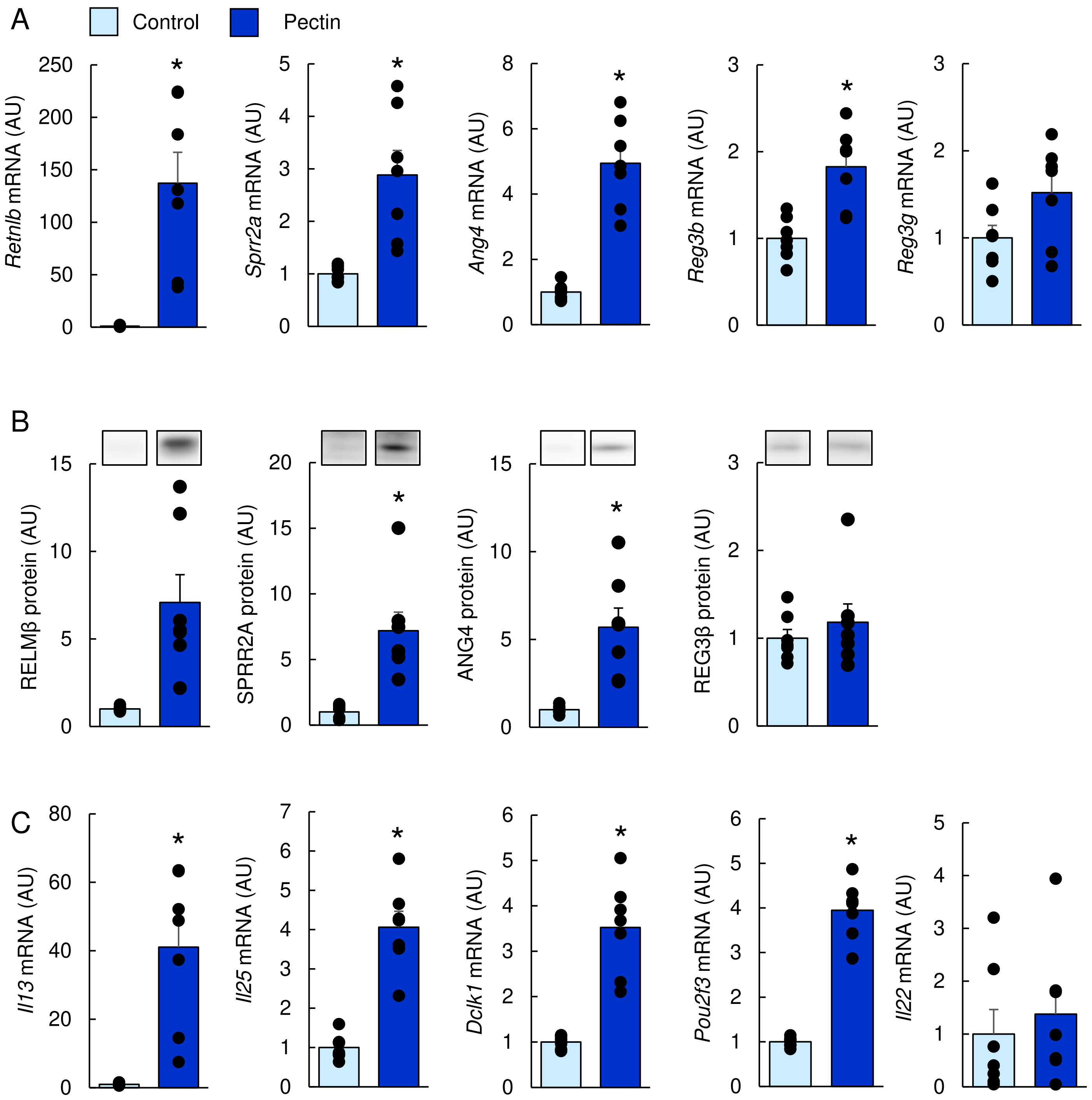

Fig. S4. Pectin supplementation upregulates antimicrobial proteins and type 2 immunity–related cytokines and expands tuft cells in the mouse ileum.

Data are from Experiment 1. Mice were fed control or 10% pectin diets for 5 days. Ileal mRNA levels of *Retnlb, Sprr2a, Ang4, Reg3g, and Reg3b* were quantified by quantitative reverse transcription-PCR (qRT-PCR; A), and protein expression of RELMβ, SPRR2A, ANG4, and REG3β was analyzed by immunoblotting (B). mRNA levels of *Il13, Il25, Dclk1, Pou2f3*, and *Il22* were quantified by qRT-PCR (C), Data are presented as mean ± s.e.m. Statistical significance was determined using Student's t-test or Mann–Whitney U test. $P < 0.05$ vs. control.

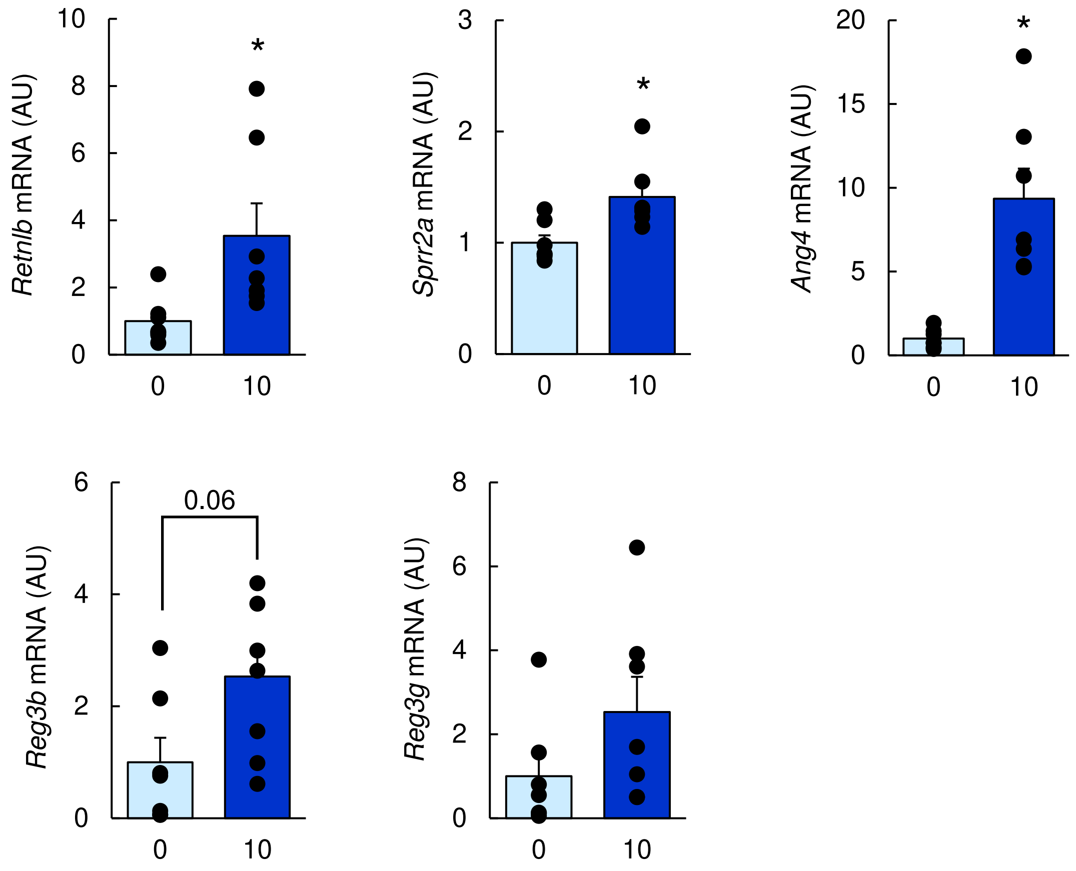

Fig. S5. Pectin supplementation upregulates antimicrobial proteins in the mouse colon.
Data are from Experiment 1. Mice were fed control or 10% pectin diets for 5 days. Colonic mRNA levels of *Retnlb, Sprr2a, Ang4, Reg3g*, and *Reg3b* were quantified by quantitative reverse transcription-PCR (qRT-PCR). Data are presented as mean ± s.e.m. Statistical significance was determined using Student's t-test or Mann–Whitney U test. *P* < 0.05 vs. control.

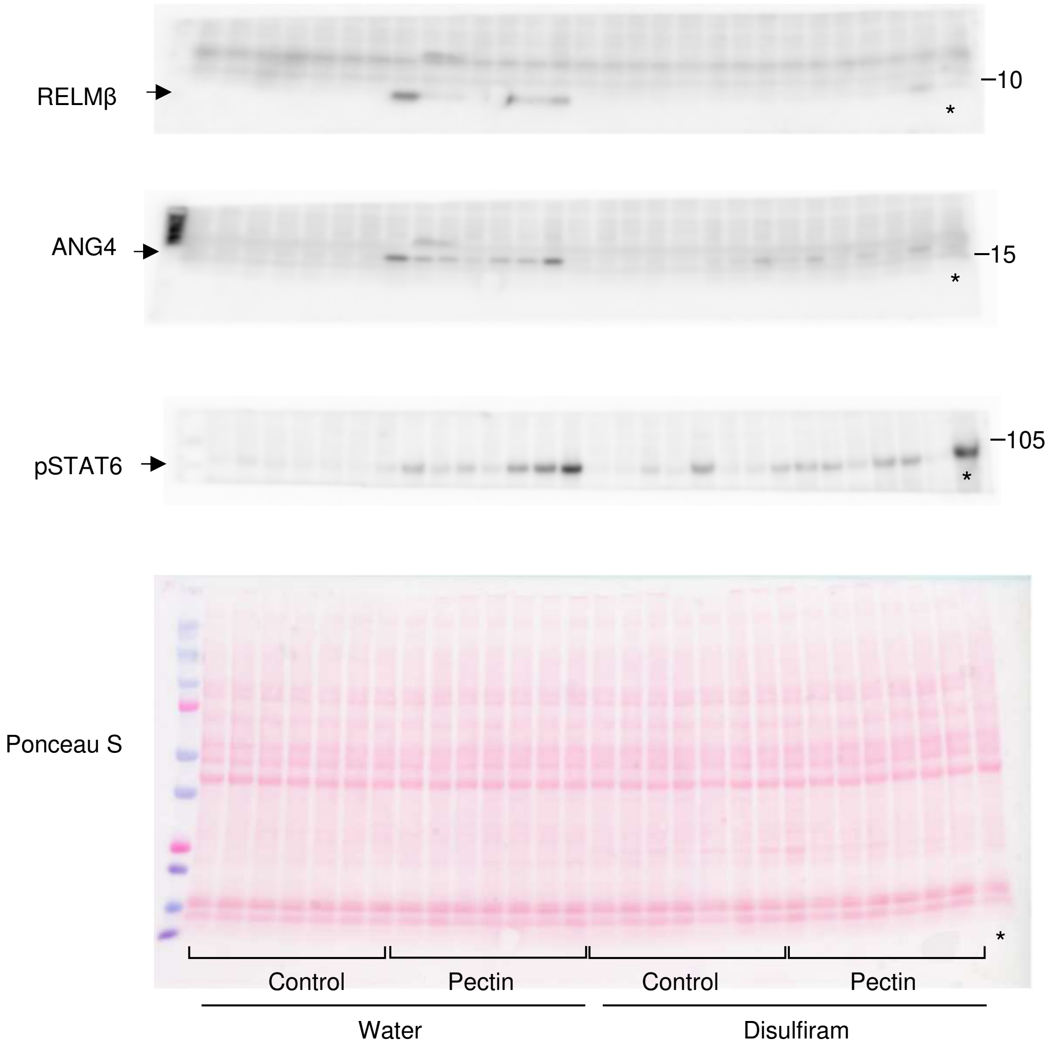

Fig. S6. Uncropped original immunoblot images corresponding to the blots shown in Fig. 4.
Data are from Experiment 3. Mice were fed control or pectin-containing diets, with or without disulfiram, for 5 days. Jejunal expression of RELMβ, ANG4, and pSTAT6 was analyzed by immunoblotting. After transfer to PVDF membranes, total protein loading was visualized by Ponceau S staining and used for normalization. Membranes were cut horizontally according to molecular weight markers and probed separately with primary antibodies. The sample marked with an asterisk is unrelated to the present experiment and was not included in the analysis.

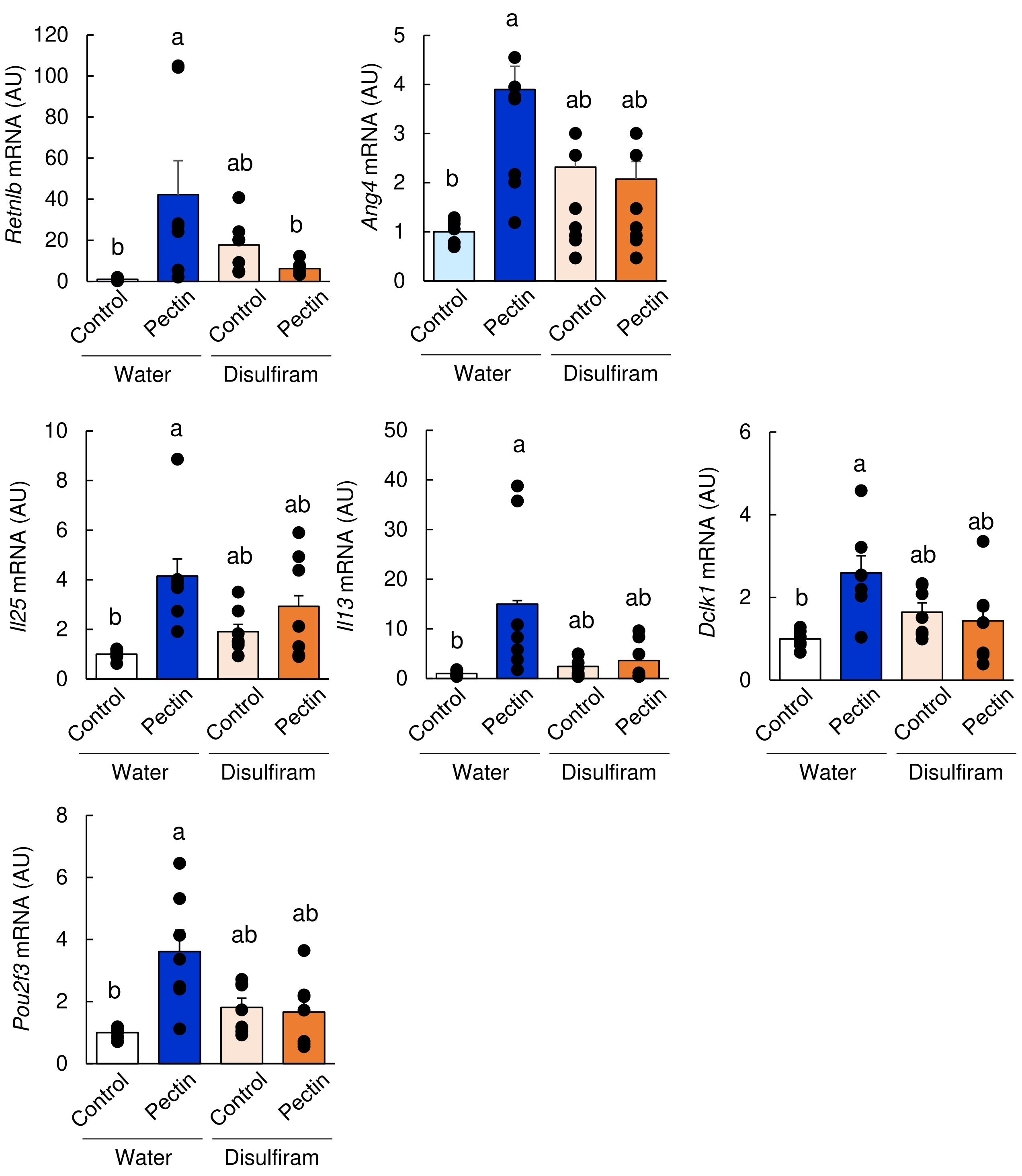

Fig. S7. ILC2 mediates pectin-induced antimicrobial protein expression, type 2 immune responses, and tuft cell expansion in the mouse ileum.

Data are from Experiment 3. Mice were fed control or pectin-containing diets with or without disulfiram for 5 days. Ileal mRNA expression levels of *Retnlb, Ang4, Il13, Il25, Dclk1*, and *Pou2f3* were quantified by quantified by quantitative reverse transcription-PCR (qRT-PCR). Statistical significance was determined using the Tukey–Kramer or Steel–Dwass test. Groups not sharing a common letter are significantly different ($P < 0.05$).

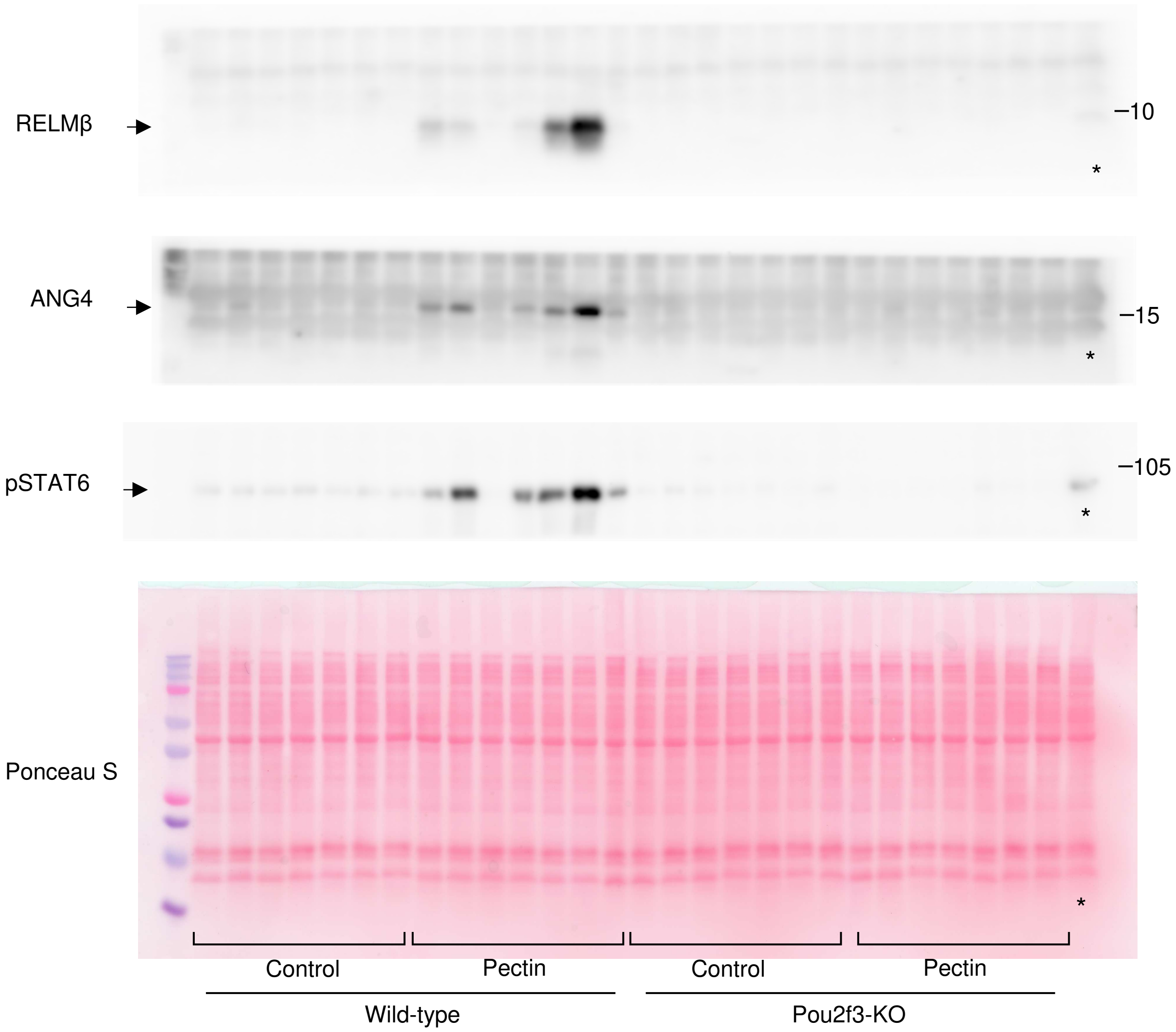

Fig. S8. Uncropped original immunoblot images corresponding to the blots shown in Fig. 6.
Data are from Experiment 4. Wild-type and Pou2f3-knockout mice were fed control or pectin-containing diets for 5 days. Jejunal expression of RELMβ, ANG4, and pSTAT6 was analyzed by immunoblotting. After transfer to PVDF membranes, total protein loading was visualized by Ponceau S staining and used for normalization. Membranes were cut horizontally according to molecular weight markers and probed separately with primary antibodies. The sample marked with an asterisk is unrelated to the present experiment and was not included in the analysis.

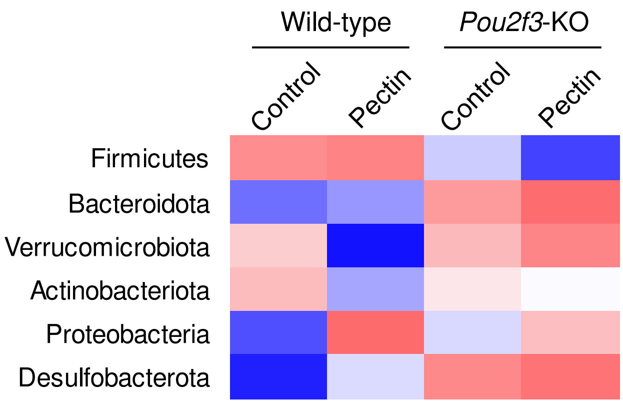

Genotype	Diet	G x D
**		
**		
**	0.06	**
*	**	0.05
0.08		

*$P<0.05$
**$P<0.01$

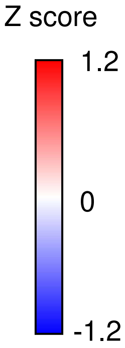

Fig. S9. Effects of pectin supplementation and tuft cell deficiency on phylum-level microbiota composition in the mouse small intestine.

Data are from Experiment 4. Wild-type and *Pou2f3*-knockout mice were fed control or pectin-containing diets for 5 days. Phylum-level microbiota composition was analyzed by 16S rRNA gene sequencing. Relative abundances are shown as a heatmap with Z-score normalization across samples. Statistical analysis was performed using two-way ANOVA to assess the effects of genotype and diet.

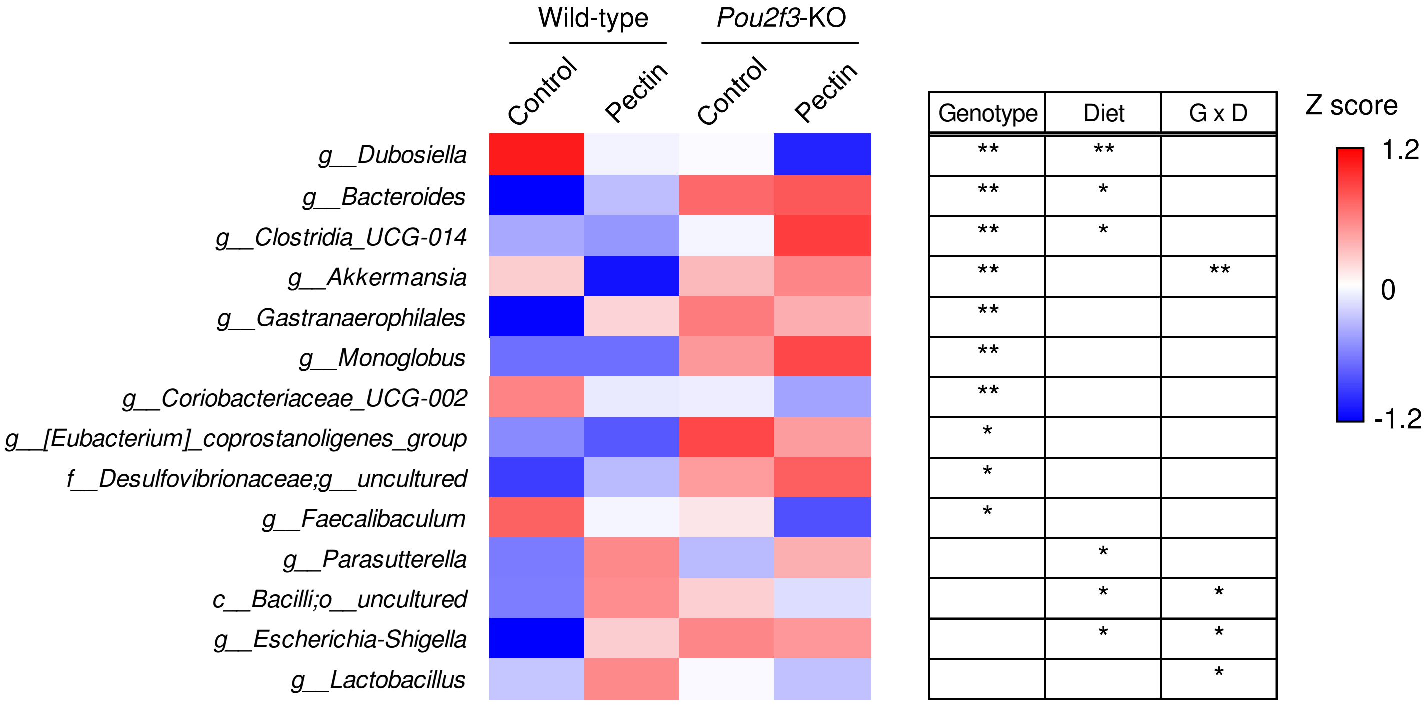

	Genotype	Diet	G x D
g__Dubosiella	**	**	
g__Bacteroides	**	*	
g__Clostridia_UCG-014	**	*	
g__Akkermansia	**		**
g__Gastranaerophilales	**		
g__Monoglobus	**		
g__Coriobacteriaceae_UCG-002	**		
g__[Eubacterium]_coprostanoligenes_group	*		
f__Desulfovibrionaceae;g__uncultured	*		
g__Faecalibaculum	*		
g__Parasutterella		*	
c__Bacilli;o__uncultured		*	*
g__Escherichia-Shigella		*	*
g__Lactobacillus			*

*$P<0.05$
**$P<0.01$

Fig. S10. Effects of pectin supplementation and tuft cell deficiency on genus-level microbiota composition in the mouse small intestine.

Data are from Experiment 4. Wild-type and *Pou2f3*-knockout mice were fed control or pectin-containing diets for 5 days. Genus-level microbiota composition was analyzed by 16S rRNA gene sequencing. Relative abundances are shown as a heatmap with Z-score normalization across samples. Statistical analysis was performed using two-way ANOVA to assess the effects of genotype and diet. Only genera showing significant effects in the two-way ANOVA are shown.

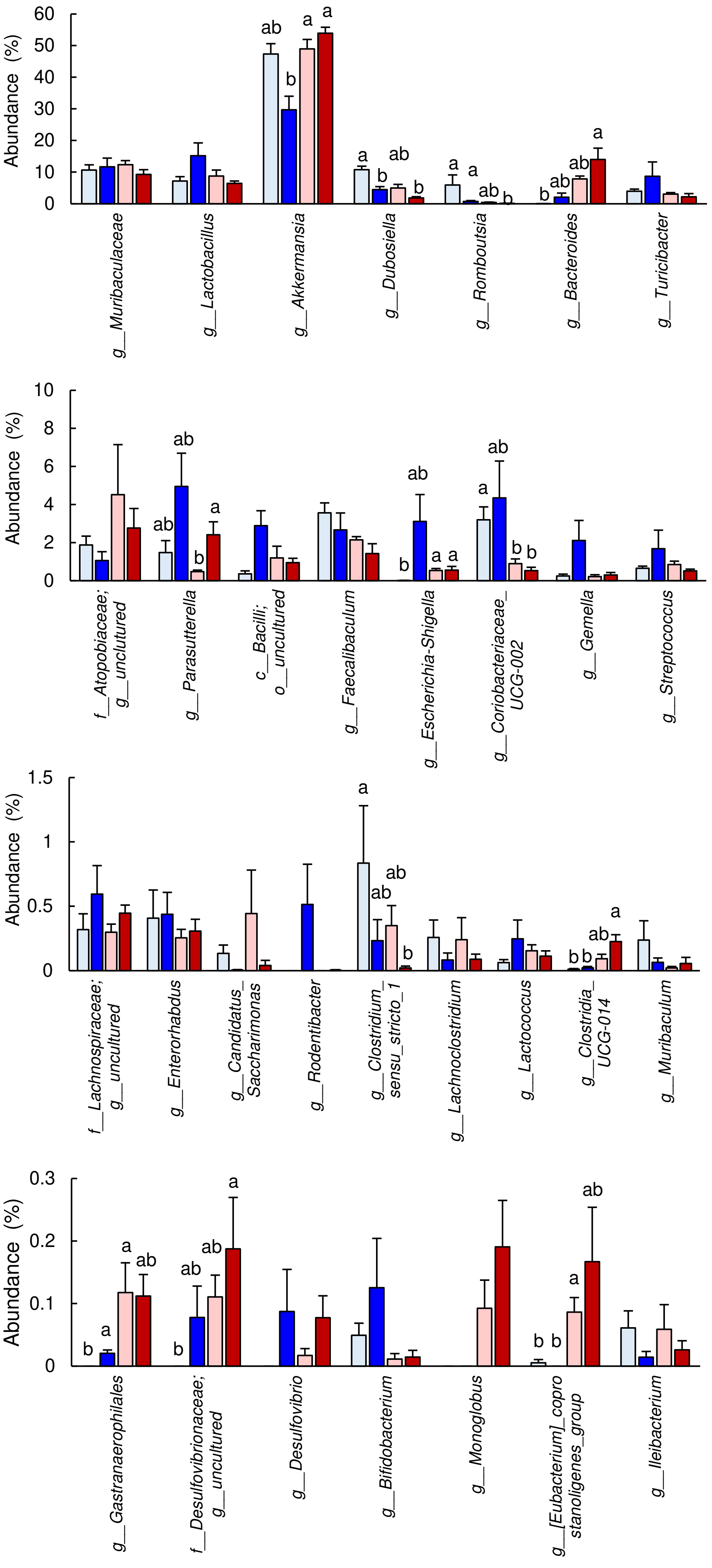

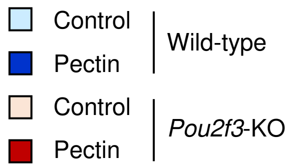

Fig. S11. Genus-level microbiota composition in the mouse small intestine.
Data are from Experiment 4. Wild-type and *Pou2f3*-knockout mice were fed control or pectin-containing diets for 5 days. Relative abundances of all detected bacterial genera are shown. Statistical significance was determined using the Tukey–Kramer or Steel–Dwass test. Groups not sharing a common letter are significantly different ($P < 0.05$).
